# Influence of Shod and Barefoot Running on the *In Vivo* Kinematics of the First Metatarsophalangeal Joint

**DOI:** 10.3389/fbioe.2022.892760

**Published:** 2022-05-16

**Authors:** Faning Zhang, Dongqiang Ye, Xini Zhang, Xiaole Sun, Shen Zhang, Shaobai Wang, Weijie Fu

**Affiliations:** ^1^ School of Kinesiology, Shanghai University of Sport, Shanghai, China; ^2^ School of Physical Education and Training, Shanghai University of Sport, Shanghai, China; ^3^ Key Laboratory of Exercise and Health Sciences of Ministry of Education, Shanghai University of Sport, Shanghai, China; ^4^ Shanghai Frontiers Science Research Base of Exercise and Metabolic Health, Shanghai University of Sport, Shanghai, China

**Keywords:** dual fluoroscopic imaging system, barefoot, shod, first metatarsophalangeal joint, *in vivo* kinematics

## Abstract

The biomechanics of the first metatarsophalangeal joint (MTPJ) is affected by different shoe conditions. In the biomechanical research field, traditional skin marker motion capture cannot easily acquire the *in vivo* joint kinematics of the first MTPJ in shoes. Thus, the present study aims to investigate the differences of the first MTPJ’s six-degree-of-freedom (6DOF) kinematics between shod and barefoot running by using a high-speed dual fluoroscopic imaging system (DFIS). In total, 15 healthy male runners were recruited. Computed tomography scans were taken from each participant’s right foot for the construction of 3D models and local coordinate systems. Radiographic images were acquired at 100 Hz while the participants ran at a speed of 3 m/s ± 5% in shod and barefoot conditions along an elevated runway, and 6DOF kinematics of the first MTPJ were calculated by 3D–2D registration. Paired sample *t*-tests were used to compare the kinematic characteristics of the first MTPJ 6DOF kinematics during the stance phase between shod and barefoot conditions. Compared with barefoot, wearing shoes showed significant changes (*p* < 0.05): 1) the first MTPJ moved less inferior at 50% but moved less superior at 90 and 100% of the stance phase; 2) the peak medial, posterior, and superior translation of the first MTPJ significantly decreased in the shod condition; 3) the extension angle of the first MTPJ was larger at 30–60% but smaller at 90 and 100% of the stance phase; 4) the maximum extension angle and flexion/extension range of motion of the first MTPJ were reduced; and 5) the minimum extension and adduction angle of the first MTPJ was increased in the shod condition. On the basis of the high-speed DFIS, the aforementioned results indicated that wearing shoes limited the first MTPJ flexion and extension movement and increased the adduction angle, suggesting that shoes may affect the propulsion of the first MTPJ and increase the risk of hallux valgus.

## 1 Introduction

Shoes are primarily used to protect the foot from injuries and improve running performance (Wolf et al., 2008; Fuller et al., 2015; Kakouris et al., 2021). In the shod condition, the foot and shoe act together to modulate the mechanical function of the first metatarsophalangeal joint (MTPJ) (Day and Hahn, 2019). In other words, the function of the first MTPJ is influenced by the shoe properties. Previous studies have found that increased midsole bending stiffness would decrease the peak extension angle and negative work of the first MTPJ (Stefanyshyn and Nigg, 2000; Roy and Stefanyshyn, 2006), thus improving the running economy. The rocker-soled shoes offered a transition with a lower extension angle and decreased peak pressure of the first MTPJ from heel strike to push off during walking (Lin et al., 2013; Menz et al., 2016). However, the effect of shoes on the first MTPJ was not beneficial. Shu et al. (2015) found that habitual barefoot runners have more straight alignment of the first MTPJ than those who are habitually wearing shoes, which suggested that shoe-wearing might increase the risk of hallux valgus. Similarly, a recent study found that compared with the barefoot condition, the medial stress of the first MTPJ was significantly increased in the shod condition, which was related to the process of hallux valgus (Yu et al., 2020). Meanwhile, the stress on the medial capsule of the first MTPJ produced by walking and running with shoes was believed to form a torque to tear the medial side of the joint capsule, which may accelerate the process of hallux valgus (Yu et al., 2020). The aforementioned inconsistent findings might be partially due to the inaccurate kinematics of the first MTPJ obtained in the shod condition.

Most of the previous studies on the first MTPJ were based on skin marker motion capture. While skin marker motion capture provides relatively efficient estimates of joint kinematics, markers move relative to the underlying skeleton, resulting in soft tissue artifacts (Roach et al., 2021). Shultz et al. (2011) found that the translational soft tissue artifact at the calcaneus in the quasi-static position was 12.1 ± 0.3 mm at the toe-off. Roach et al. (2021) found that the positions of the fifth metatarsal skin marker were significantly different from the DFIS-tracked virtual markers during loading and unloading, which may indicate that skin marker motion capture is less accurate during quicker motions. To obtain the joint kinematics more precisely, some studies have carried out experiments by digging holes in shoe surfaces (Wegener et al., 2015), which could eliminate the effect of shoe surfaces, but still could not acquire the real skeletal motion in shod conditions. Although some researchers quantified the first MTPJ movement using intracortical pins (Arndt et al., 2013), this method is invasive and susceptible to infection and may disturb normal movement. In addition, previous studies often focused only on the sagittal movement but fail to explore the transverse movement of the first MTPJ. It may not fully understand the joint kinematics characteristics. Therefore, more accurate techniques should be used to obtain the kinematics of the first MTPJ during shod running.

A high-speed dual fluoroscopic imaging system (DFIS), a noninvasive medical imaging measurement technology, compensates for the limitations of traditional biomechanical methods by dynamically capturing *in vivo* joint motion without being affected by the relative movement of the skin and other soft tissues (Ye et al., 2021). The measuring precision of the DFIS in determining the joint position and capturing the six-degree-of-freedom (6DOF) motion in bony structures is on the sub-millimeter and sub-degree level, which is suitable for exploring the kinematic characteristics of the foot joint (Cross et al., 2017).

The purpose of this study therefore is to investigate the 6DOF kinematic difference of the first MTPJ between shod and barefoot running using the high-speed DFIS. We hypothesized that shoe-wearing limited the 6DOF of the first MTPJ and specifically increased the peak adduction angle in the horizontal plane compared with barefoot.

## 2 Materials and Methods

### 2.1 Participants

In total, 15 healthy male runners (age: 30.9 ± 7.3 years, height: 172.7 ± 4.4 cm, weight: 70.3 ± 8.4 kg, weekly running volume: 46.4 ± 23.4 km) were recruited. The inclusion criteria were as follows: 1) habitual rear-foot strike runners, 2) right-foot dominant, 3) running more than 20 km per week, and 4) no lower limb injury in the past 6 months. This study was approved by the Institutional Review Board of the Shanghai University of Sport (No. 102772021RT034).

### 2.2 Instrumentation

#### 2.2.1 Computed Tomography

The 64-row 128-slice spiral CT (SOMATOM, Germany) was used to take the CT images of the subjects’ feet at the neutral position of the right foot. The scanning layer thickness and spacing were all 0.6 mm; the voltage was 120 kV; the current was 140 mA; the length, width, and height of the voxel were set as 0.488, 0.488, and 0.625 mm, respectively; and the size of the voxel was 512 × 512 × 256.

#### 2.2.2 High-Speed DFIS

**FIGURE 1:**
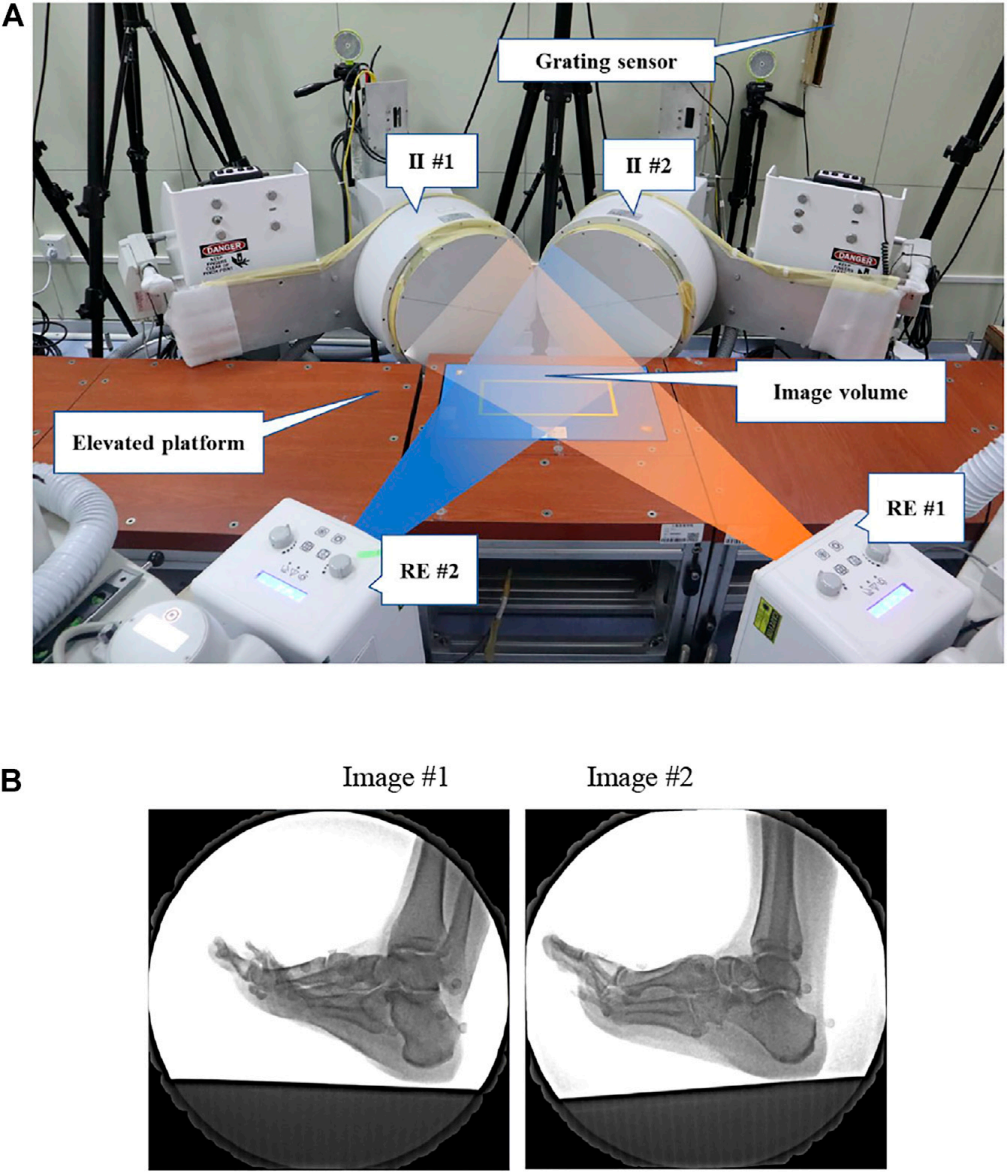
**(A)** High-speed DFIS setup. Participants ran on an elevated platform. The image intensifiers (II #1 and II #2) processed images created by X-rays from the radiographic emitters (RE #1 and RE #2). **(B)** DFIS images showed the first MTPJ of a participant.

The high-speed DFIS consisted of two pairs of fluoroscopic imaging systems, which were, respectively, composed of two fluorescence emitters that generate X-rays and two image intensifiers that receive and image X-rays, with a diameter of 431.8 mm (Figure 1). In this study, the distances between the two fluorescence emitters and the image intensifiers were 132.2 and 128.6 cm, respectively, and the two image intensifiers were positioned at 119.6° to one another. The shooting voltage was 60 kV, the current was 63 mA, the shooting frequency was 100 Hz, the exposure speed was 1/1,000 s, and the image resolution was 1,024 × 1,024 pixels.

#### 2.2.3 Witty–Manual Grating Timing System

A wireless Witty–Manual grating timing system (Microgate, Bolzano, Italy) was used to guarantee the target running speed.

#### 2.2.4 Grating Sensor

An infrared blocking grating sensor (GJ-2004, Changju Electronic Technology, China) was applied to trigger the DFIS while the participants ran through the image volume.

### 2.3 Data Collection

The participants were asked to wear experimental vests, shorts, and shoes (traditional footwear, heel-to-toe drop: 6 mm; midsole material: TPU, EVA; upper structure: textile fabric; toe box: width, 11 cm, depth, 8 cm, height, 3 cm, size, 9; without any arch support) before the running experiment. Then, the participants warmed up for 5 min on a treadmill at a speed of 3 m/s. Before initiating the measurement process, several practice trials were performed, so that the participants became familiar with the elevated runway. Radiographic images were acquired at 100 Hz while the participants ran at a speed of 3 m/s ± 5% in shod and barefoot conditions along the elevated runway with their right foot landing within the image volume naturally. One successful trial was collected using high-speed DFIS in each condition (Campbell et al., 2016; Welte et al., 2021), which was guided by the X-ray image quality.

### 2.4 Data Processing

#### 2.4.1 CT Scans and Reconstruction of the First MTPJ

**FIGURE 2:**
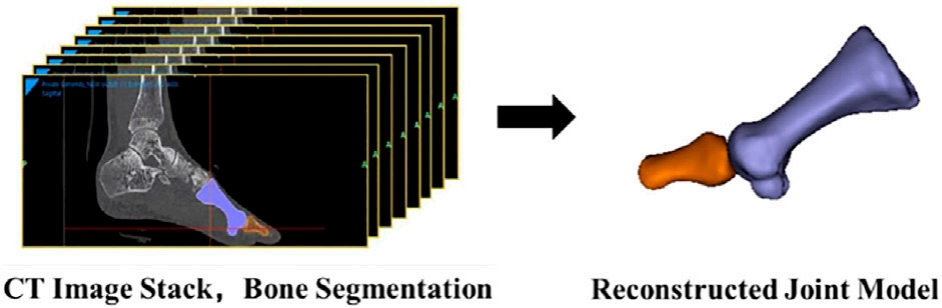
Reconstruction of the first MTPJ.

CT scans were taken from each participant’s right foot while they were supine with a neutral ankle position. The first metatarsal and first proximal phalanx were segmented and reconstructed first using the MTPJ bone models in mimics (v. 21.0; Mimics, Materialise, Leuven, Belgium, Figure 2).

#### 2.4.2 Establishment of a Coordinate System

Inertial anatomical coordinate systems were generated from the bone meshes, with the origin located at the centroid and the x–y–z-axes aligned along the principal axes of the moment of inertia tensor (Eberly D et al., 1991). A rectangular box was fitted around the first metatarsal that touched the bone model contours in the superior–inferior, medial–lateral, and anterior–posterior directions. It can decrease potential variability in the identification of bony landmarks (Esfandiarpour et al., 2018). The box vertex coordinates were used to calculate the geometric center and the axis of symmetry by the plug-in of the Rhinoceros (v6.0, McNeel & Associates, Seattle, United States). The geometric center of the box was considered the origin of the coordinate system. Moreover, the first metatarsal was considered a symmetrical rigid body of uniform mass, so the axis of symmetry of the skeleton was the principal axis of the moment of inertia tensor. Y-axis was along the principal axis (Eberly D et al., 1991), and x-axis and z-axis were perpendicular to the y-axis, respectively. The x-axis was lateral–medial, the y-axis was anterior–posterior, and the z-axis was superior–inferior (Welte et al., 2021). The coordinate system of the first proximal phalanx was established in the same way.

#### 2.4.3 Definition of 6DOF Kinematics

**FIGURE 3:**
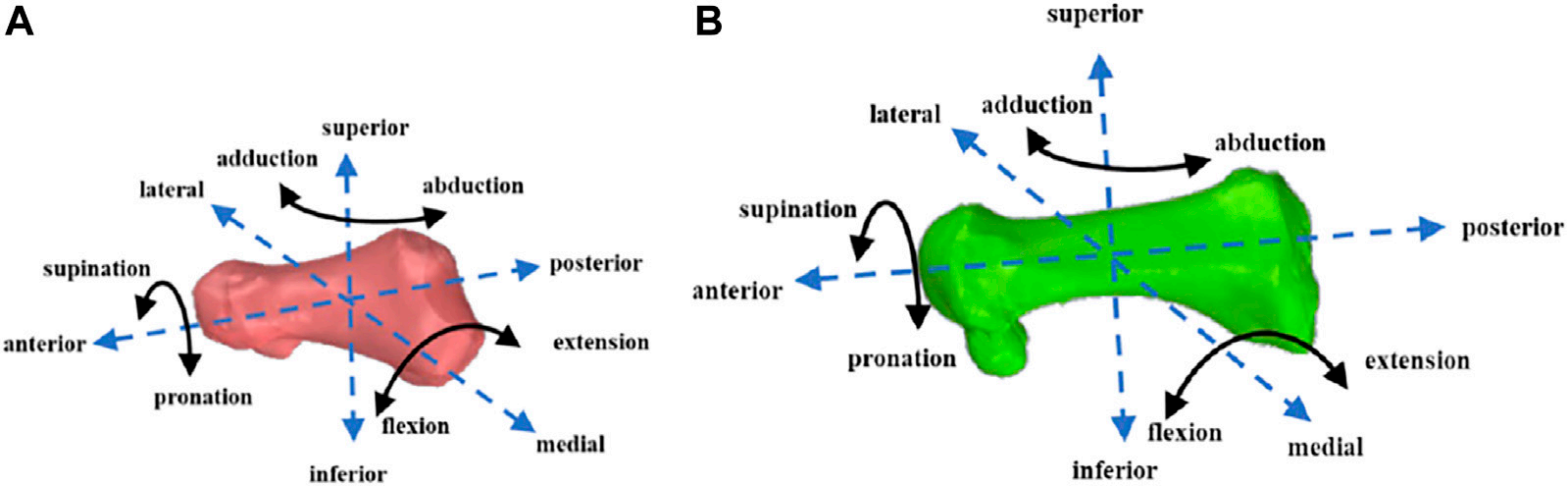
First proximal phalanx **(A)** and first metatarsal **(B)** motion diagram.

The 6DOF kinematics of the first MTPJ was defined as the relative motions of the first metatarsal coordinate system with respect to the first proximal phalanx coordinate system. The medial/lateral, anterior/posterior, and superior/inferior directions were aligned with the x-, y-, and z-axes of the coordinate systems, respectively (Welte et al., 2021). Extension/flexion (EX/FL), supination/pronation (SUP/PRO), and abduction/adduction (AB/AD) were determined as rotations around the medial/lateral, anterior/posterior, and superior/inferior axes, respectively (Figure 3). The positive values represented anterior translation, lateral translation, superior translation, EX, SUP, and AB, and the negative values corresponded to the opposite.

#### 2.4.5 *In Vivo* Kinematics

Images obtained with high-speed DFIS are subject to distortion when X-ray beams are transformed into visible images (Tersi and Stagni, 2014). Pincushion distortion and magnetic lens distortion are the two major sources of distortion (Wang and Blackburn, 2000). To correct for the distortion, an X-ray image was collected with an “un-distortion” grid (Brainerd et al., 2010). The grid consisted of a perforated piece of aluminum plate with the known size and spacing of each hole. A software program, XMALab, used a distortion-correcting algorithm to correct for any changes to the spacing or size of the holes in the perforated grid (Rohr et al., 2001).

**FIGURE 4:**
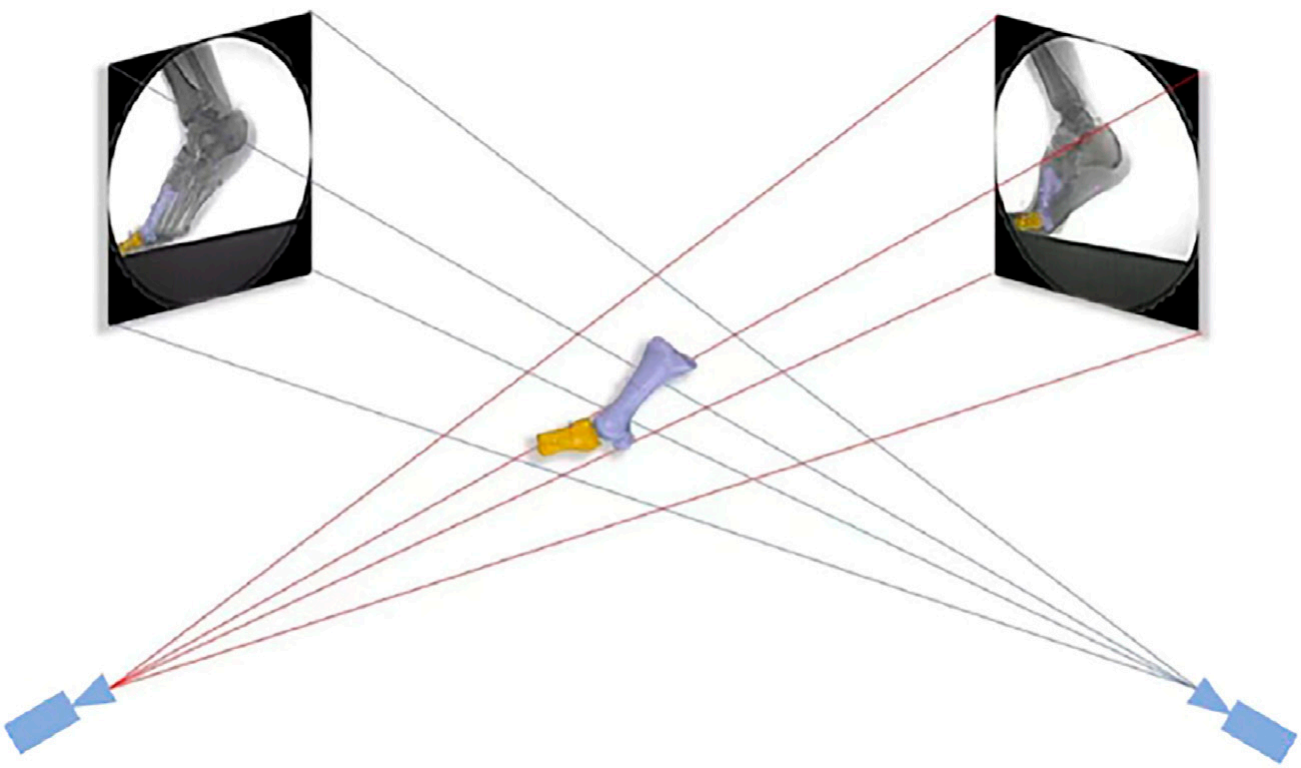
3D–2D registration. The position of the joint was adjusted by translating and rotating the joint model in the software until the edge of the bones matched the radiographic images.

Calibrated fluoroscopic images were then imported into the modeling software (Rhinoceros 6.0, McNeel & Associates, Seattle, United States). The 3D models were also imported into the same software in the virtual 3D environment and were moved and rotated independently at increments of 0.01 mm and 0.01° (Cao et al., 2019). The kinematic results of the first MTPJ were calculated by 3D–2D registration (Figure 4). The specific indicators include the range of motion (ROM) at 6DOF, which was defined as the maximum minus the minimum joint angle among all frames; initial contact angle; and peak angle in each DOF. The stance phase was divided into 10 sections with 10% per section. The kinematics of each counterpart section were compared.

### 2.5 Statistics

The mean and standard deviation for each variable were calculated. The paired sample *t*-test was used to compare the 6DOF data of the first MTPJ under two conditions (SPSS 25.0, IBM, Chicago, United States). The significance level was set as α = 0.05.

## 3 Results

### 3.1 *In Vivo* Kinematics of the First MTPJ

#### 3.1.1 Joint Translation

**FIGURE 5:**
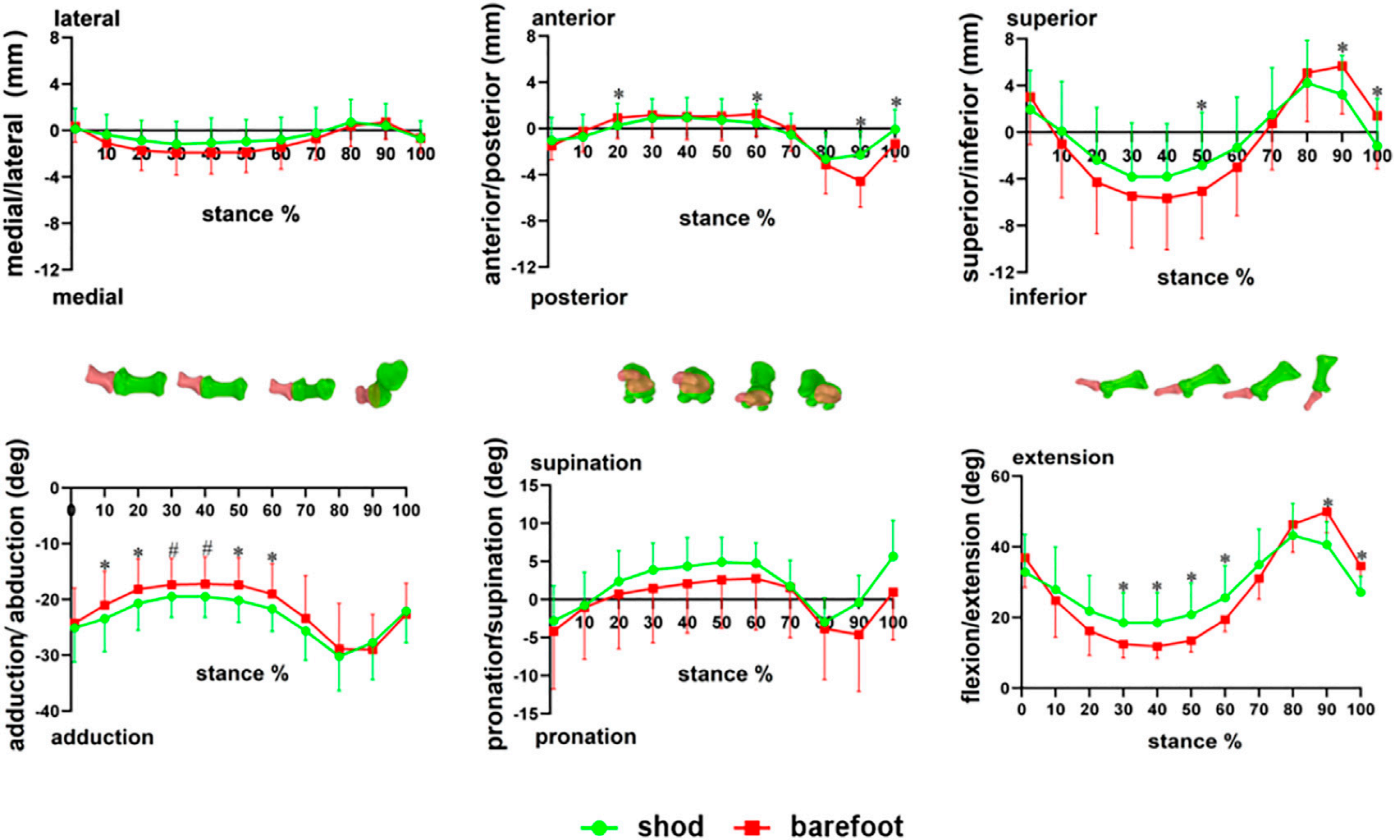
Six degrees of freedom of the first MTPJ during stance. * there are significant differences between the shod and barefoot conditions, *p* < 0.05; ^#^ there are different trends between the shod and barefoot conditions, *p* < 0.1.

Compared with barefoot, the first MTPJ moved less inferior at 50% (*p* = 0.032) but moved less superior at 90% (*p* = 0.014) and 100% (*p* = 0.007) of the stance phase in the shod condition. The anterior translation of the first MTPJ at 20 and 60% and the posterior translation at 90–100% of the stance phase were significantly reduced in the shod condition (*p* < 0.05, Figure 5).

#### 3.1.2 Joint Rotation

Compared with barefoot, the EX angle of the first MTPJ was larger at 30–60% but smaller at 90–100% of the stance in the shod condition (*p* < 0.05). The AD angle of the first MTPJ was larger at 10, 20, 50, and 60% of the stance period in the shod condition (*p* < 0.05). There were no significant differences in PRO and SUP of the first MTPJ during the stance phase (Figure 5).

### 3.2 Angles and ROM of the First MTPJ

#### 3.2.1 Joint Translation

**FIGURE 6:**
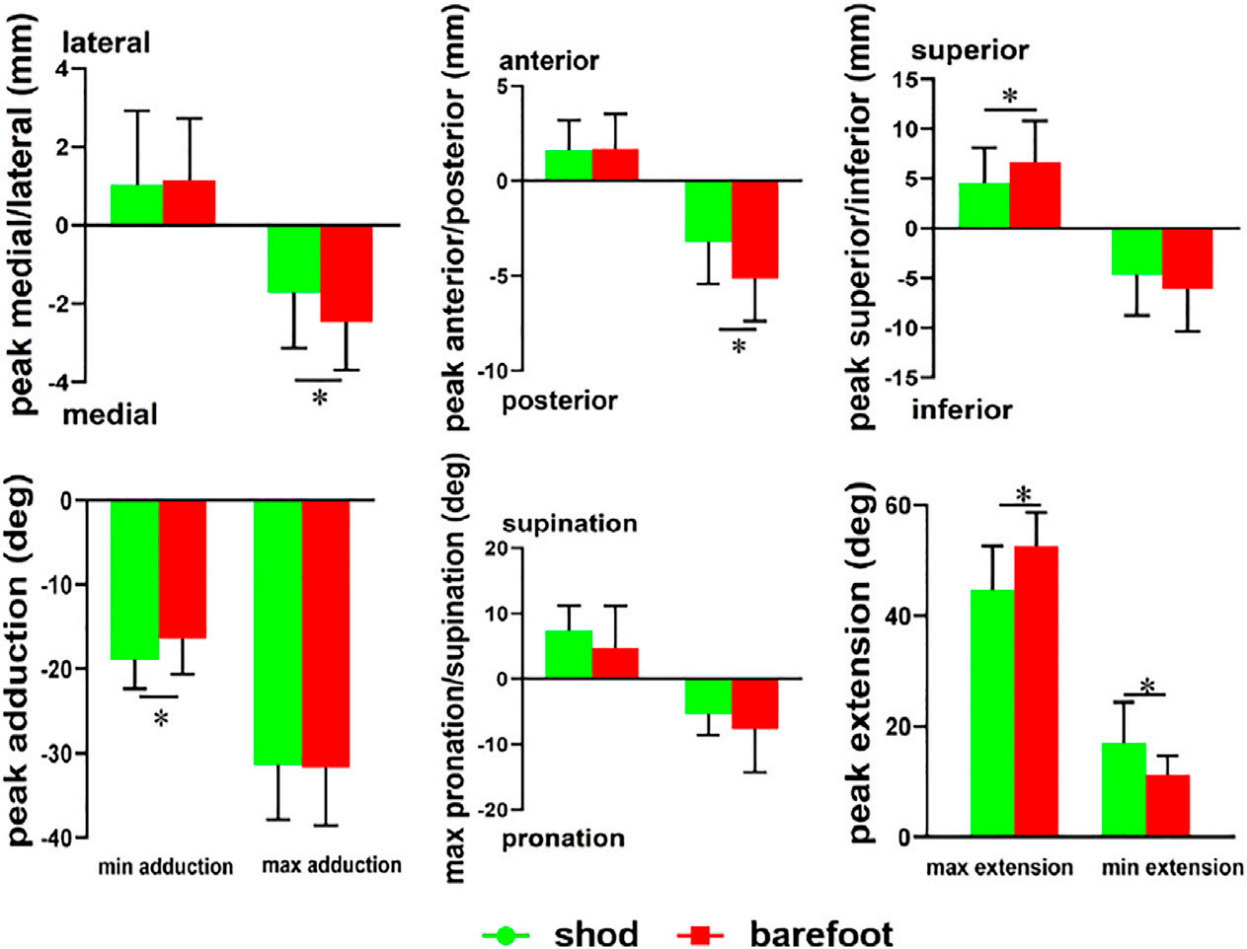
Peak angle of the first MTPJ in shod and barefoot. * compared with barefoot, there are significant differences in the shod condition, *p* < 0.05.

During the stance phase, the first MTPJ’s peak medial (*p* = 0.039), posterior (*p* < 0.001), and superior movement (*p* = 0.043) (Figure 6); anterior to posterior ROM (*p* = 0.002) and superior to inferior ROM (*p* < 0.001) were significantly smaller in the shod condition than in the barefoot condition (Table 1).

**TABLE 1:** Comparison of translation, rotation at initial contact, and ROM of the first MTPJ in shod and barefoot.

	Condition	M/L (mm)	A/P (mm)	S/I (mm)	EX/FL (°)	PRO/SUP (°)	AB/AD (°)
Initial contact	Barefoot	−0.34 ± 1.35	−1.49 ± 1.21	3.03 ± 4.1	36.94 ± 8.47	−4.16 ± 7.62	−24.26 ± 6.3
	Shod	0.14 ± 1.76	−1.04 ± 1.99	1.96 ± 3.34	32.82 ± 10.61	−2.8 ± 4.58	−25.09 ± 6.1
ROM	Barefoot	3.6 ± 1.26	6.82 ± 2.37^a^	12.78 ± 2.47^a^	41.36 ± 6.77^a^	12.35 ± 3.29	15.34 ± 4.62
	Shod	2.76 ± 0.98	4.81 ± 2.27	9.22 ± 2.55	27.68 ± 5.48	12.82 ± 4.07	12.48 ± 4.55

a compared with barefoot, there are significant differences in the shod condition, *p* < 0.05. M/L: medial/lateral translation; A/P: anterior/posterior translation; S/I: superior/inferior translation; EX/FL: extension/flexion; PRO/SUP: pronation/supination; AB/AD: abduction/adduction; ROM: range of motion; “+”: the first MTPJ, medial, anterior, superior translation and extension, supination, abduction; “−”: the first MTPJ, lateral, posterior, inferior translation and flexion, pronation, and adduction.

#### 3.2.2 Joint Rotation

Compared with barefoot, the maximum EX of the first MTPJ was smaller (*p* < 0.001), whereas the minimum EX and AD were significantly larger (*p* = 0.009) in the shod condition (Figure 6). Meanwhile, the FL/EX ROM in the shod condition was significantly smaller (*p* < 0.001), whereas there were no significant differences in the PRO/SUP and AD/AB ROM (Table 1).

## 4 Discussion

This study found that compared with barefoot, the peak medial/posterior/superior translation, the maximum EX angle, and the ROM of the EX were significantly reduced in the shod condition, whereas the minimum EX and AD angle were increased. These results were consistent with the hypothesis that shoe-wearing restricted partial 6DOF movement of the first MTPJ.

The first MTPJ in the sagittal plane was significantly reduced in the shod condition, which was consistent with previous studies (Lin et al., 2013; Wegener et al., 2015; Sichting et al., 2020). Specifically, Wegener et al. (2015) found that shoes reduced the first MTPJ’s peak EX during running from 31.5° (barefoot) to 12.6° (shod), and Sichting et al. (2020) found that the EX ROM of the first MTPJ decreased from 27.9 (barefoot) to 19.7 (shod). The minimum EX angle of the first MTPJ in the shod condition was significantly increased. It may partially be due to the toe spring of the footwear, which increased the minimum EX angle during the stance phase (Sichting et al., 2020). In addition, runners exhibited significantly greater medial longitudinal arch compression in the barefoot condition than in the shod condition (Holowka et al., 2022). Thus, the plantarflexion of the first metatarsal was decreased, which might cause the minimum EX angle of the first MTPJ to increase in the shod condition. These results based on skin marker motion capture were consistent with the results of this study which shows that shoes restricted the EX motion of the first MTPJ. However, the minimum and maximum EX angles during the stance phase were larger than those in previous studies using skin marker motion capture. In the study of McDonald et al. (2016), who used skin marker motion capture, the minimum EX of the first MTPJ was close to 0° in barefoot and shod running at 2.7 m/s, whereas the maximum EX was 34.2 ± 3.9° (barefoot) and 30.1 ± 2.8° (shod), which were far smaller than the maximum angle (shod: 44.7 ± 7.9°; barefoot: 52.6 ± 6.0°) in the current study. Midfoot plantarflexion, primarily the first metatarsal, during propulsion is believed to occur due to the windlass mechanism (Wegener et al., 2015). The larger EX angle in the current study might be the result of the plantarflexion of the first metatarsal. However, traditional motion capture methods cannot easily detect the *in vivo* motion of the first metatarsal, thus failing to obtain the most accurate motion of the first MTPJ. In addition, the EX of the first MTPJ plays an important role in calculating joint dynamics, and the underestimation of FL/EX activity by traditional motion capture methods may have affected the joint dynamic results of previous studies. However, the current research based on high-speed DFIS is still at an early stage. Moreover, we did not calculate the dynamics in this study. Therefore, future studies should compare the differences in the dynamic results of the first MTPJ obtained by DFIS and other motion capture methods.

This study showed that the peak superior translation of the first MTPJ was significantly reduced under the shod condition, which may be related to the anatomical structure of the first MTPJ. Human metatarsal heads are dorsally oriented and mediolaterally broad articular surfaces. The dorsally oriented metatarsal heads in the human forefoot are thought to increase the range of EX motion at the MTPJ by providing more dorsal articular surface area on which the proximal phalangeal base can slide (Fernandez et al., 2016; Sichting et al., 2020) In the current study, the EX trend of the first MTPJ was consistent with the superior/inferior translation trend, which further supported the result of the previous study (Phillips et al., 1996). The shoe limited the flexion and extension of the first MTPJ, thus reducing the ROM of joint translation in the sagittal plane.

In the current study, the AD angle of the first MTPJ in the static CT model of all participants was within 15°–20°, which was consistent with the clinical standard for mild hallux valgus (Coughlin et al., 2002; Xiang et al., 2018). Previous studies have shown that the distance between the first and second MTPJs was generally narrower in habitually shod runners than in habitually barefoot runners. The straight alignment between the first metatarsal and the first proximal phalangeal was disrupted, so habitually shod runners have a higher risk of suffering from hallux valgus (Shu et al., 2015). The first MTPJ straight alignment of participants in this study may have been affected by long-term shoe-wearing running. In addition, the AD angle of the first MTPJ in shod was significantly greater than that in barefoot, which was consistent with our hypothesis. Using finite element simulation, Yu et al. (2020) found that with the increase in the AD angle of the first MTPJ, the stress on the medial joint capsule increased, and the stress on the medial joint capsule was significantly higher under shoe-wearing than barefoot. The positive and negative axial principal stresses formed a combined torque in the medial capsule of the first MTPJ. The medial capsule was damaged by the torque during periodic movement in shod, such as running, indicating that shoe-wearing may induce hallux valgus. By contrast, the first MTPJ had a decreased AD angle and increased AD/AB ROM under the barefoot condition, which meant that the forefoot has more space for movement. Thus, the joint motion in the horizontal plane was increased. Barefoot running could potentially help improve the shape and function of the first MTPJ (D’AoÛt et al., 2009) and then may contribute to the correction of hallux valgus. Although this study was cross-sectional research, the previous study showed that barefoot training could improve the hallux valgus. Xiang et al. (2018) performed a 12-week five-finger shoe running intervention for 15 subjects with mild and moderate hallux valgus and found that the degree of hallux valgus was significantly reduced after the intervention. The five-finger shoe, without any support or cushioning, only protects the skin of the foot and is thought to mimic barefoot conditions (Smith et al., 2015; Holowka et al., 2018). While footwear helps the foot resist external injuries, its relatively small toe space not only limits the movement of the first MTPJ but also makes the medial side subject to external torque during propulsion, potentially accelerating the occurrence and progression of hallux valgus.

In this study, high-speed DFIS provided a new perspective for analyzing the *in vivo* motion characteristics of the first MTPJ during running, but there were still some limitations: only male participants who were used to running in shoes were recruited, and gender differences were not explored. Due to ionizing radiation, only data on one valid aspect were collected. The experimental shoes were traditional running shoes, and the kinematic differences of the first MTPJ under different toe sizes should be explored in future studies. In addition, future studies should further explore the effect of training on the *in vivo* kinematics of the first MTPJ during running by using DFIS.

## 5 Conclusion

On the basis of a high-speed DFIS, the 6DOF kinematic difference of the first MTPJ in the whole stance phase was investigated, which created a more accurate measurement method for studying the movement of the small joint of the foot. The results showed that shoe-wearing limited the extension and joint translation of the first MTPJ and increased the horizontal adduction angle, suggesting that shoes could limit the propulsion effect and ROM of the first MTPJ and might increase the risk of hallux valgus. This finding indicated that shoemakers should increase the capacity of the forefoot movement to lower the risk of injury.

## Data Availability

The raw data supporting the conclusion of this article will be made available by the authors, without undue reservation.
